# Supplementary material for: Ranking environmental degradation trends of plastic marine debris based on physical properties and molecular structure
Source: Nat Commun. 2020 Feb 5;11:727. doi: 10.1038/s41467-020-14538-z (PMC7002677; doi:10.1038/s41467-020-14538-z)
Supplement: Supplementary file 1 — Supplementary Information [file 41467_2020_14538_MOESM1_ESM.pdf]

Supplementary Information: Ranking Environmental Degradation Trends of Plastic Marine Debris Based on Physical Properties and Molecular Structure

Min et al.

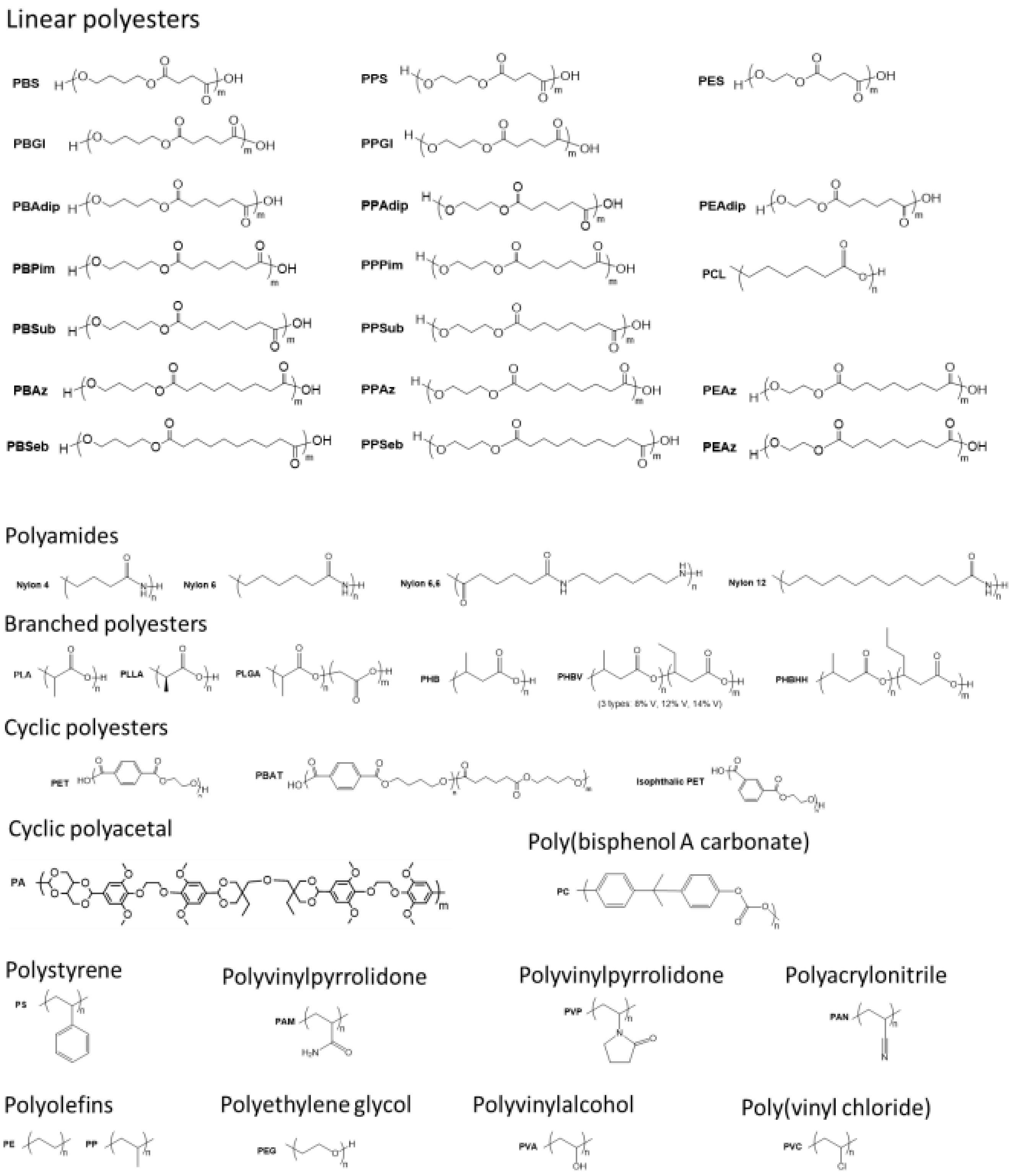

Supplementary Figure 1. Partial list of polymer structures in database.

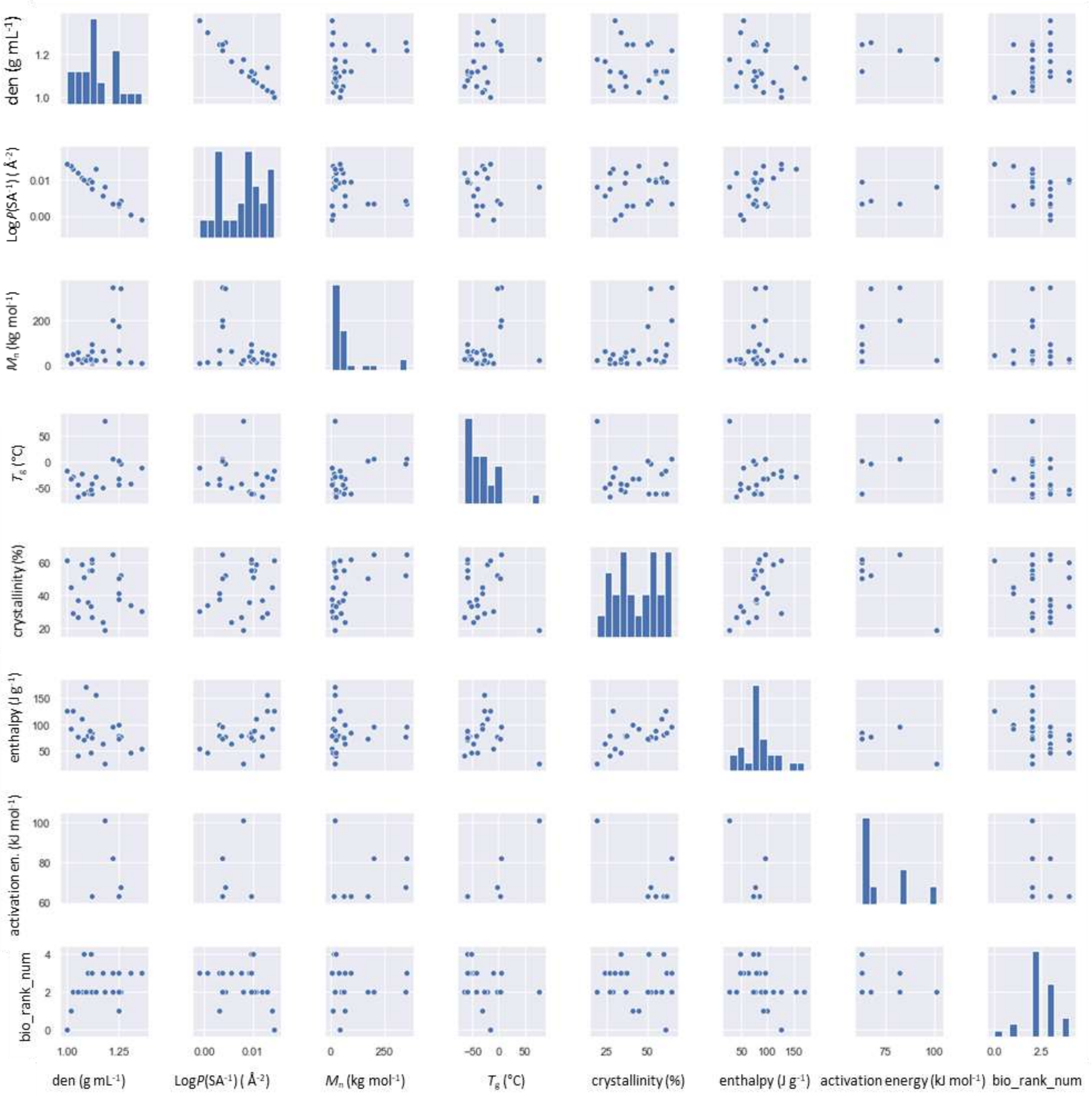

Supplementary Figure 2. Partial example of initial screening of database variables.

Variables with high correlation, such as density-Log$P$(SA)$^{-1}$ and enthalpy-crystallization, were noted to reduce the total number of features for machine learning.

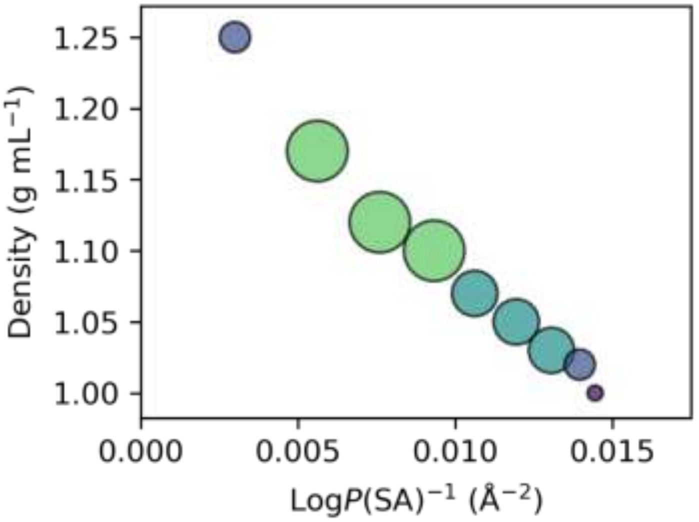

Supplementary Figure 3. Correlation between computational Log*P*(SA)$^{-1}$ values and density for polyesters. Size of circles and colors indicate rate of biodegradation (large light green = fast, cyan = medium, purple = slow, violet = very slow) from literature.[1]

a

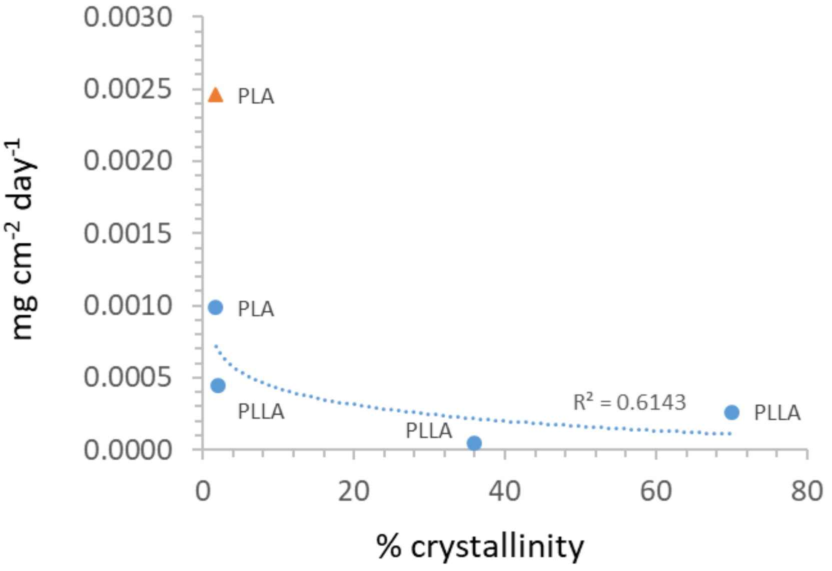

b

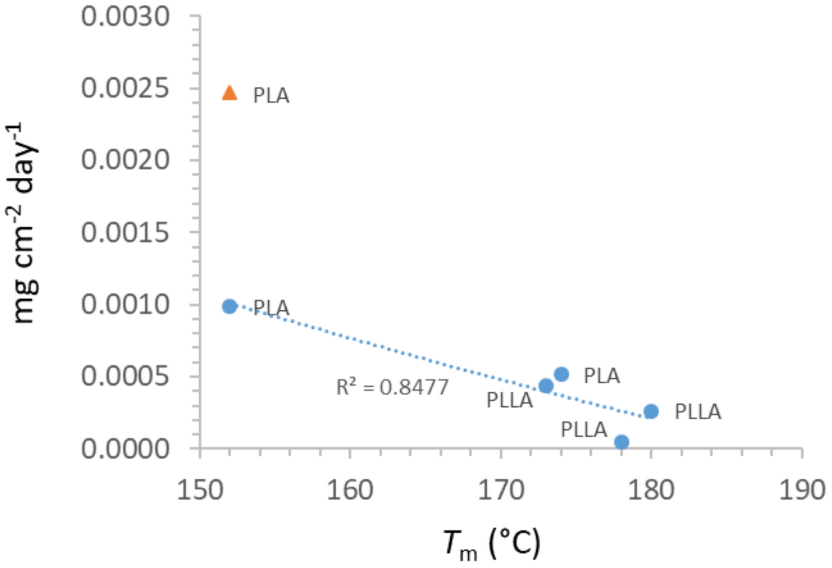

Supplementary Figure 4. Influence of crystallinity on surface erosion of PLA and PLLA. a) % crystallinity and **b**) melting point. Data shown for 25 °C (blue circles) and 40 °C (orange triangles). Data lines represent logarithmic regression.

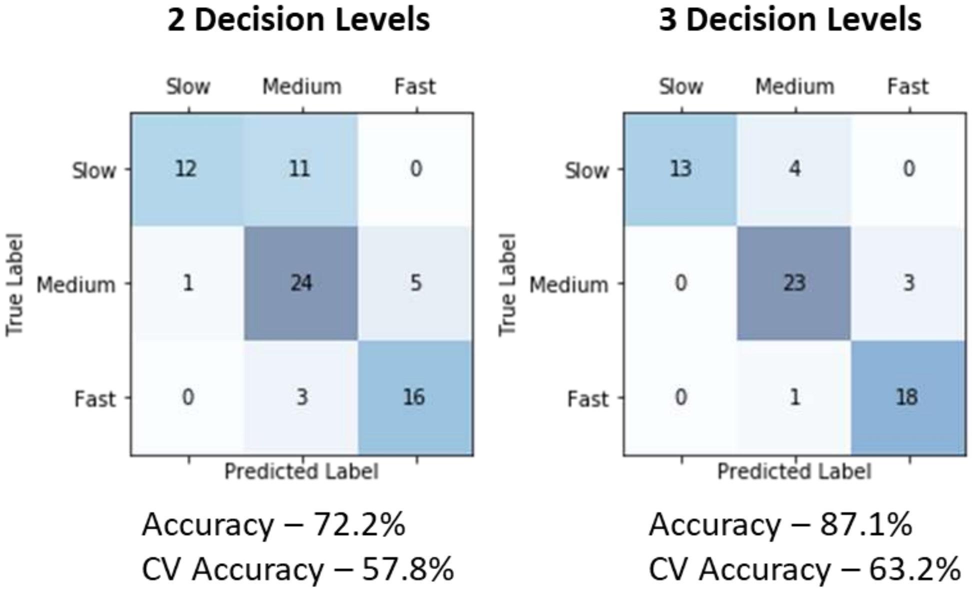

Supplementary Figure 5. Prediction of accuracy for classification trees via confusion matrices. These matrices compare predictions (i.e. Predicted Label on x-axis) with the actual category (i.e. True Label on y-axis). For instance, out of the 19 plastics that were assigned as fast, the 2-level tree predicted 16 of these as fast and 3 as medium for an accuracy of 72.2 %. In contrast, the 3-level tree predicted 18 of these as fast and 1 as medium for an accuracy of 87.1 %. The CV (i.e. cross-validation) accuracy represents a 10-fold cross-validation procedure using stratified sampling.

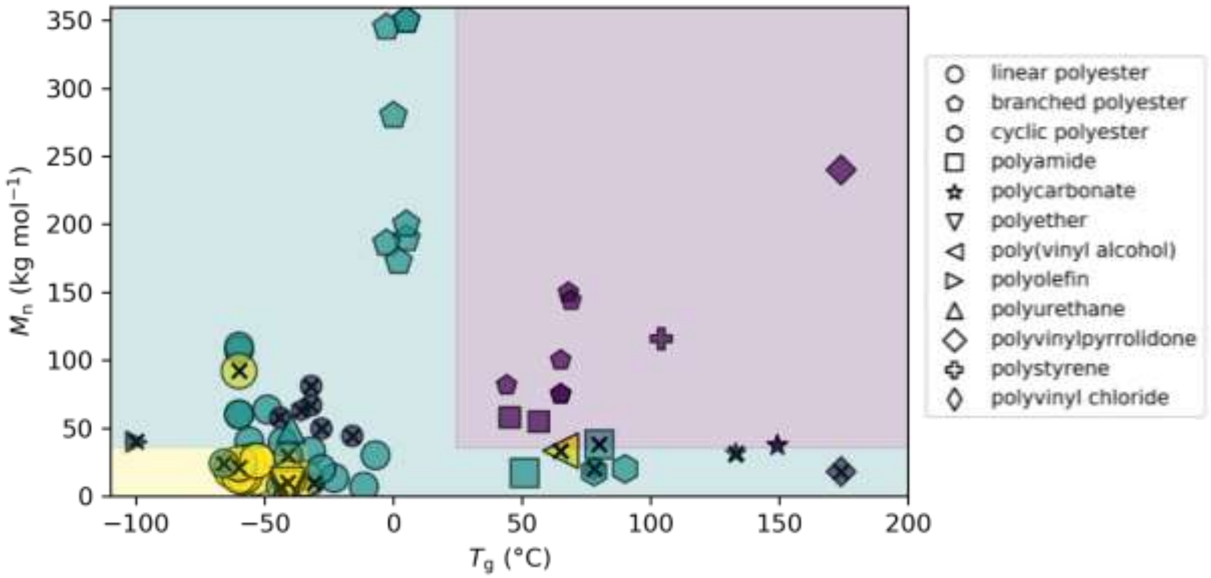

Supplementary Figure 6. Graphical representation of 2-level decision tree. Data shown for biotic processes in Figure 4 based on molecular weight and T_g. The shaded area correspond to fast (yellow), medium (cyan), and slow (violet) degradation. Incorrect predictions are show with a black ‘x’.

a

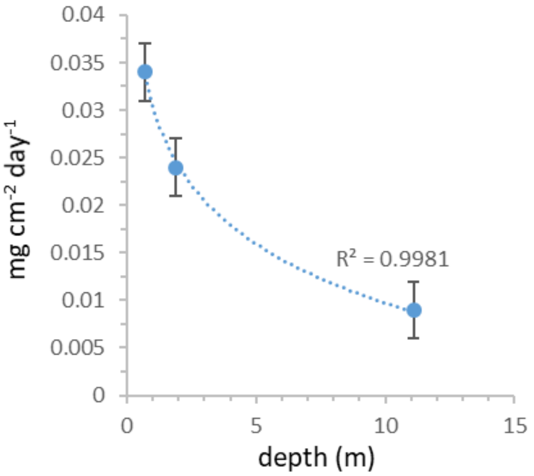

b

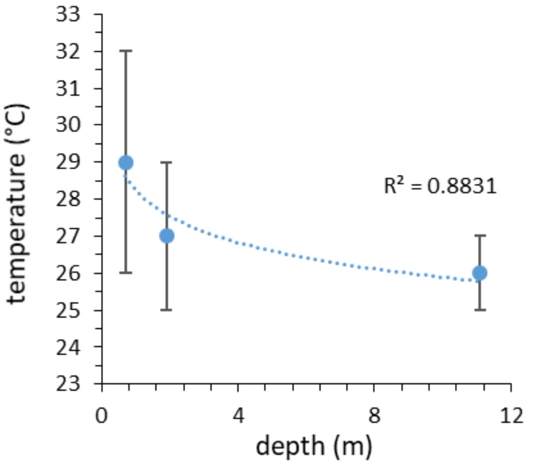

Supplementary Figure 7. Analysis of data for PHBV copolymers.[2] Influence of depth on **a**) surface erosion and **b**) average temperature values. Error bars represent temperature fluctuation for each depth.

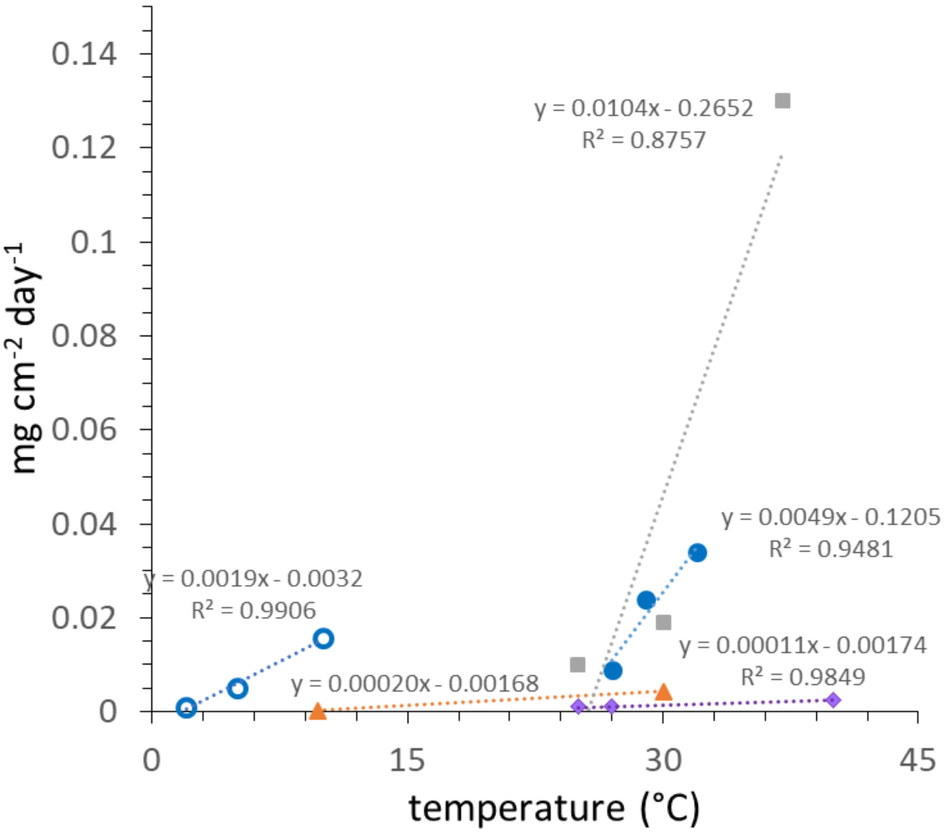

Supplementary Figure 8. Effect of water temperature on surface erosion. Data shown for PCL (grey squares), PHBV in deep sea conditions (open blue circles),[3] PHBV in coastal area (solid blue circles),[2] Nylon 6 (orange triangles),[4,5] and PLA (purple diamonds).[6,7] Dotted lines represent linear regression equations.

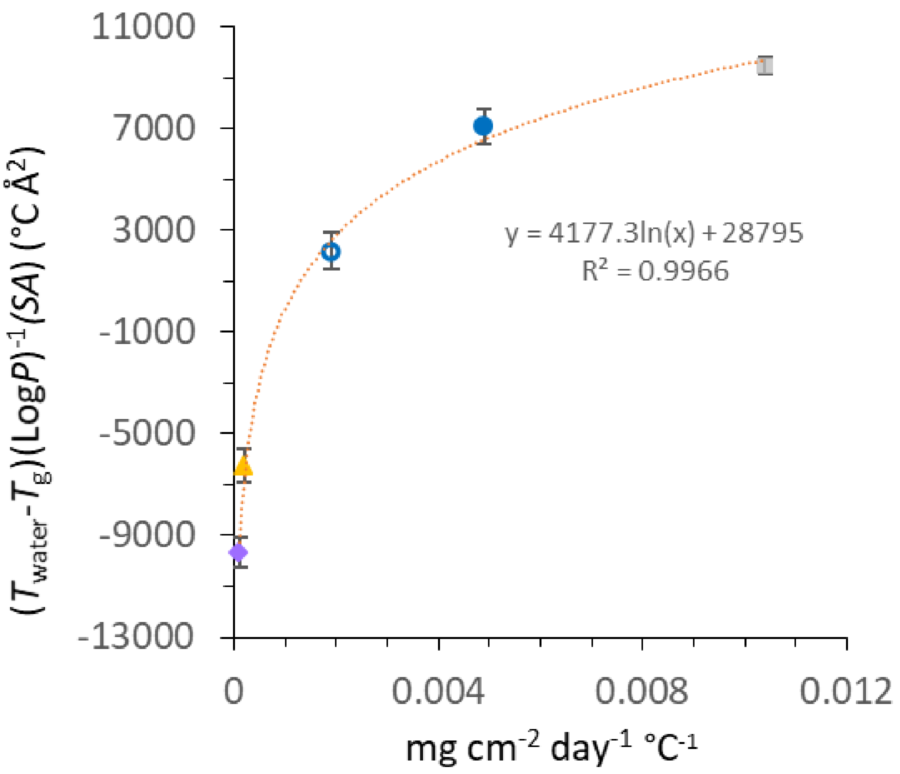

Supplementary Figure 9. Modeling surface erosion data. Comparison of average water temperature (T_{water}), T_g and Log$P$(SA)$^{-1}$ on y-axis with rate of surface erosion (mg cm^{-2} day^{-1} °C^{-1}) on x-axis. Values for x-axis taken from slope of regression data in Supplementary Data 8. Data shown for PCL (grey square), PHBV in deep sea conditions (open blue circle), PHBV in coastal area (solid blue circle), Nylon 6 (orange triangle), and PLA (purple diamond). Error bars represent the range of y-axis values for a ±3 °C temperature fluctuation in T_{water}.

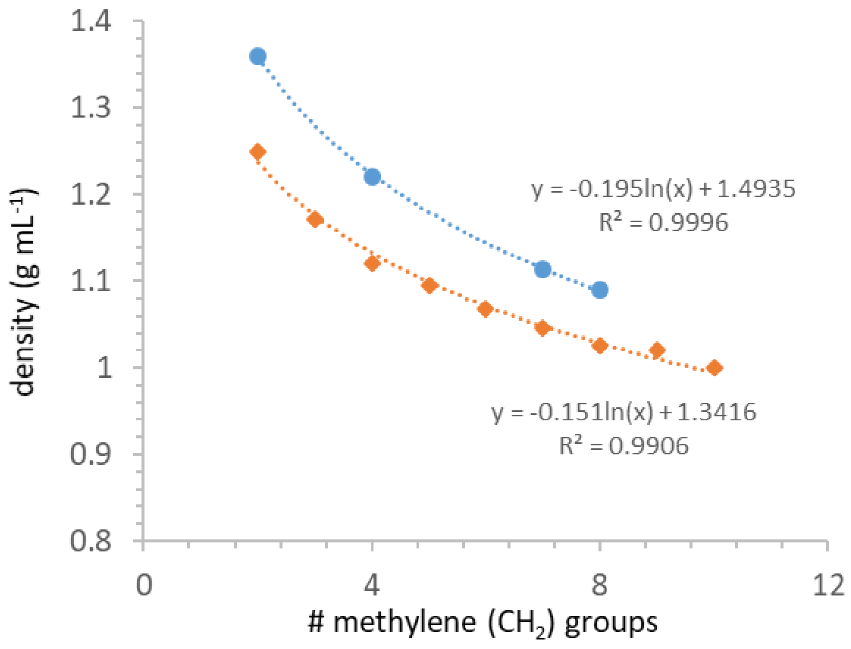

Supplementary Figure 10. Density for polyesters made via polycondensation. Orange diamonds represent data for 1,4-butanediol-based polyesters. Blue circles represent data for ethylene glycol-based polyesters. Values on x-axis correspond to number of methylene groups in dicarboxylic acid.

Plastic	Structure	T_g (°C)	Log$P$(SA)$^{-1}$ (Å^{-2})	Predicted rate (k) using Equation 2 (mg cm^{-2} day^{-1} °C^{-1})	Relative degradation rate (k_{rel})
PCL	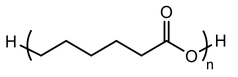	-60	0.0096	0.00658	160.2
PU (MDI-PTHF)	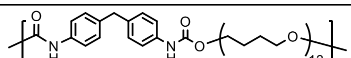	-38	0.00697	0.00626	152.4
PHBV	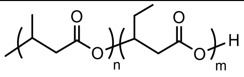	-2	0.0043	0.00261	63.6
PU (MDI-BD)	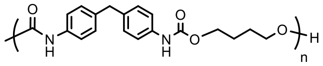	90	0.0122	0.000233	5.7
PC	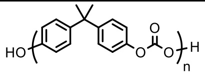	149	0.0213	0.000225	5.5
Nylon 6	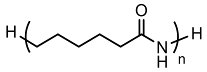	48	0.0045	0.000175	4.3
PET	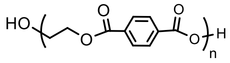	78	0.00807	0.000157	3.8
PLA	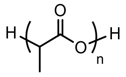	60	0.00336	0.0000411	1.0

Supplementary Table 1. Predicted surface erosion based on Equation 2. Relative degradation rates are given for plastics in seawater at 15 °C.

Plastic	Structure	Location	E_{total} (mg cm^{-2} day^{-1})	Predicted rate (k) using Equation 2 (mg cm^{-2} day^{-1} °C^{-1})	T_{water} (°C)	b	E_{waves} (mg cm^{-2} day^{-1})
PHBV	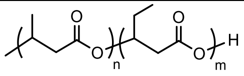	Coastal	0.034	0.00570	29	-0.148	0.017
PHBV	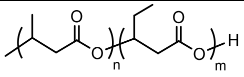	Estuary	0.020	0.00603	30	-0.166	0.005

Supplementary Table 2. Surface erosion based on Equation 3 for polyesters in seawater.

Supplementary References

1 Baba, T., Tachibana, Y., Suda, S. & Kasuya, K.-i. Evaluation of environmental degradability based on the number of methylene units in poly(butylene n-alkylenedionate). *Polym. Degrad. Stabil.* **138**, 18-26 (2017).

2 Imam, S. H. *et al.* Degradation of starch-poly(beta-hydroxybutyrate-co-beta-hydroxyvalerate) bioplastic in tropical coastal waters. *Appl. Environ. Microbiology* **65**, 431-437 (1999).

3 Sekiguchi, T. *et al.* Biodegradation of aliphatic polyesters soaked in deep seawaters and isolation of poly(ε-caprolactone)-degrading bacteria. *Polym. Degrad. Stabil.* **96**, 1397-1403 (2011).

4 Friedrich, J., Zalar, P., Mohorčič, M., Klun, U. & Kržan, A. Ability of fungi to degrade synthetic polymer nylon-6. *Chemosphere* **67**, 2089-2095 (2007).

5 Welden, N. A. & Cowie, P. R. Degradation of common polymer ropes in a sublittoral marine environment. *Mar. Pollut. Bull.* **118**, 248-253 (2017).

6 Deroiné, M. *et al.* Seawater accelerated ageing of poly(3-hydroxybutyrate-co-3-hydroxyvalerate). *Polym. Degrad. Stabil.* **105**, 237-247 (2014).

7 Sashiwa, H., Fukuda, R., Okura, T., Sato, S. & Nakayama, A. Microbial Degradation Behavior in Seawater of Polyester Blends Containing Poly(3-hydroxybutyrate-co-3-hydroxyhexanoate) (PHBHHx). *Mar. Drugs* **16**, 34-45 (2018).
