# Supplementary material for: Ranking environmental degradation trends of plastic marine debris based on physical properties and molecular structure
Source: Nat Commun. 2020 Feb 5;11:727. doi: 10.1038/s41467-020-14538-z (PMC7002677; doi:10.1038/s41467-020-14538-z)
Supplement: Supplementary file 3 — Description of Additional Supplementary Files [file 41467_2020_14538_MOESM3_ESM.pdf]

Description of Additional Supplementary Files

File Name: Supplementary Code 1
Description: The python code for machine learning

File Name: Supplementary Code 2
Description: Construction of Figures 2-5
